# The role of alkyl chain length in the melt and solution crystallization of paliperidone aliphatic prodrugs

**DOI:** 10.1107/S2052252523009582

**Published:** 2024-01-01

**Authors:** An Chen, Peishan Cai, Yayun Peng, Minshan Guo, Yuan Su, Ting Cai

**Affiliations:** a Department of Pharmaceutics, School of Pharmacy, China Pharmaceutical University, Nanjing 211198, People’s Republic of China; b Department of Pharmaceutical Engineering, School of Engineering, China Pharmaceutical University, Nanjing 211198, People’s Republic of China; University of Iowa, USA

**Keywords:** paliperidone, prodrugs, alkyl chain lengths, crystallization kinetics, crystal engineering, crystal morphology, intermolecular interactions, pharmaceutical solids

## Abstract

Fatty acid-derivative prodrugs have been utilized extensively to improve the physicochemical, biopharmaceutical and pharmacokinetic properties of active pharmaceutical ingredients. However, to our knowledge, the crystallization behavior of prodrugs modified with different fatty acids has not been explored. In the present work, a series of paliperidone aliphatic prodrugs with alkyl chain lengths ranging from C4 to C16 was investigated with respect to crystal structure, crystal morphology and crystallization kinetics. The paliperidone derivatives exhibited isostructural crystal packing, despite the different alkyl chain lengths, and crystallized with the dominant (100) face in both melt and solution. The rate of crystallization for paliperidone derivatives in the melt increases with alkyl chain length owing to greater molecular mobility. In contrast, the longer chains prolong the nucleation induction time and reduce the crystal growth kinetics in solution. The results show a correlation between difficulty of nucleation in solution and the interfacial energy. This work provides insight into the crystallization behavior of paliperidone aliphatic prodrugs and reveals that the role of alkyl chain length in the crystallization behavior has a strong dependence on the crystallization method.

## Abstract 2

Structural determination and crystallization behavior of paliperidone prodrugs modified with different alkyl chain lengths is described.

## 1. Introduction

A prodrug is a molecule with little or no pharmacological activity on its own but can be converted to an active substance through a chemical or enzymatic process or a combination of the two in the body (Rautio *et al.*, 2018 ▸; Fattahi *et al.*, 2020 ▸; Huttunen *et al.*, 2011 ▸). Prodrugs have been widely utilized in drug delivery to improve the physicochemical, biopharmaceutical and/or pharmacokinetic properties of the parent drug (Fattahi *et al.*, 2020 ▸; Markovic *et al.*, 2019 ▸). Several long-acting injectable formulations based on the prodrug strategy have been approved by the US Food and Drug Administration (FDA) for the treatment of schizophrenia, including fluphenazine enanthate, fluphenazine decano­ate, haloperidol decano­ate, paliperidone palmitate and aripiprazole lauroxil, whereby prodrugs were applied to reduce solubility and slow dissolution to achieve extended drug release (Remenar, 2014 ▸; Shi, Lu *et al.*, 2021 ▸).

Fatty acids, a class of essential compounds for the maintenance of health and normal physiological development, have received much attention in prodrug synthesis owing to their excellent biocompatibility (Fattahi *et al.*, 2020 ▸; Sun *et al.*, 2017 ▸; Sprecher, 1981 ▸; Dijkstra, 2008 ▸). The commonly used strategy is to conjugate the carboxyl end of the lipid with a hydroxyl group of the drug to form a stable ester linkage. It has been reported that physicochemical properties, such as melting point, solubility, dissolution, permeability, metabolic stability and self-assembly behavior, and biological activities including cell cytotoxicity, side effects and pharmacokinetic characteristics of prodrugs can be affected by the chain length and degree of substitution of fatty acids covalently linked to the parent drug (Zhang *et al.*, 2016 ▸; Zhong *et al.*, 2018 ▸; Thanki *et al.*, 2019 ▸; Singh *et al.*, 2021 ▸; Im *et al.*, 2011 ▸).

Crystallization is an essential process in the purification and production of substances in the pharmaceutical, agrochemical and fine chemical industries. The crystallization process comprises two stages: nucleation and crystal growth, both of which have a decisive influence on the properties of the crystalline product, such as crystalline form, purity, crystal habit, particle size distribution. In-depth understanding of the mechanism facilitates the design of the crystallization of crystalline products with desired properties. One option is to investigate the relationship between the molecular structure and crystallization behavior in a series of model analogs systematically (Cruz-Cabeza *et al.*, 2017 ▸; Tang *et al.*, 2021 ▸; Shi, Xu *et al.*, 2021 ▸; Powell *et al.*, 2014 ▸; Yang *et al.*, 2014 ▸; Kakkar *et al.*, 2020 ▸; Huang *et al.*, 2018 ▸). It has been reported in glycine homopeptides that longer chains prolonged the induction time (Guo *et al.*, 2023 ▸). Several alkyl-derivative compounds with shorter alkyl chain lengths present a reduced crystallization tendency (Honda *et al.*, 2021 ▸; Ishikawa *et al.*, 2022 ▸; Zheng *et al.*, 2017 ▸). Intuitively, a more flexible compound is more difficult to crystallize. Khan & Sundararajan (2011 ▸, 2013 ▸) reported that the spherulite size and growth rate of bis­carmates varied with the alkyl side chain length; however, the growth kinetics increase first and then decrease significantly with increasing chain length. To our knowledge, the impact of alkyl chain length on crystallization behavior of fatty acid-derivative prodrugs has not been explored, despite their extensive utilization in physicochemical, biopharmaceutical and pharmacokinetics.

**Figure 1:**
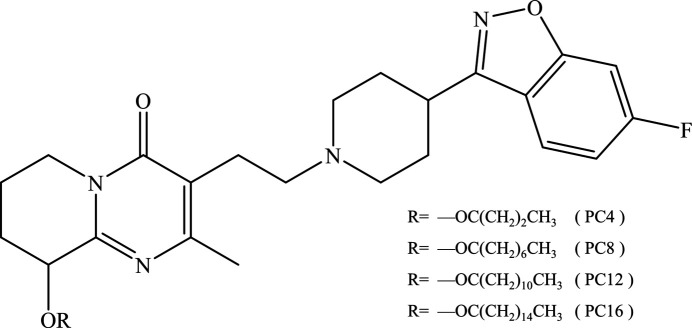
Chemical structure of paliperidone derivatives.

In the present study, a series of derivatives of the antipsychotic drug paliperidone has been selected for the model compounds (Fig. 1 ▸). The fatty acids covalently linked with paliperidone only vary in alkyl chain length, ranging from C4 to C16, hence we can explore the link between alkyl chain length and crystallization behavior. For the first time, we solved the crystal structures of the paliperidone aliphatic derivatives, including paliperidone palmitate, the active pharmaceutical ingredient of INVEGA HAFYERA, by single-crystal X-ray diffraction (SCXRD). Moreover, the crystal morphology, nucleation and crystal growth kinetics of the paliperidone derivatives were investigated in both melt and solution environments.

## 2. Experimental

### 2.1. Materials

Paliperidone butyrate (PC4), paliperidone caprylate (PC8), paliperidone laurate (PC12) and paliperidone palmitate (PC16) were purchased from Zhuzhou Focus Pharmaceutical Technology Co. Ltd, China (purity >99%). All solvents were obtained from Nanjing Chemical Reagent Co. Ltd, China (purity >95%). All materials were used without further purification.

### 2.2. Solid-state characterization

Differential scanning calorimetry (DSC) was performed using Q2000 instruments (TA, USA). Samples (3–5 mg) were enclosed in sealed aluminium pans and heated to a specified temperature under a nitro­gen atmosphere (with a fixed flow rate of 50 ml min^−1^). The instrument was calibrated with indium. To determine the glass transition temperature (*T*_g_), all paliperidone derivatives were heated to 130°C for 3 min, equilibrated to −60°C, and then reheated to the melting point of the derivatives (Fig. S1 of the supporting information).

Thermogravimetric analysis (TGA) was carried out with a TA Q500 instrument (TA, USA) to determine the thermal stability of the sample. In brief, samples (5–15 mg) were added to a platinum plate and heated to 500°C at a rate of 20°C min^−1^ under a 60 ml min^−1^ nitro­gen purge. The thermal data were analyzed using the *TA Universal Analysis* software.

Powder X-ray diffraction (PXRD) was carried out on a SmartLab XE diffractometer (Rigaku, Japan) with Cu *K*α radiation. Approximately 30 mg samples were placed on glass supports and exposed to radiation (3 ≤ 2θ ≤ 40°) at a scan rate of 10° min^−1^.

SCXRD was carried out on a Bruker D8 venture diffractometer (Bruker, USA) using Mo *K*α radiation. Unit-cell determination, diffraction data collection, integration and refinement were achieved using the program *SAINT*. *SHELX*2014/7 (Sheldrick, 2015 ▸) was used to solve and refine the structure by direct methods and full-matrix least-squares on the *F* ^2^ procedure. The data were corrected for the effects of absorption by a multiscan method using *SADABS*. Non-hydrogen atoms were refined anisotropically, and hydrogen atoms were located in geometrically calculated positions with the riding model.

### 2.3. Melt crystallization of paliperidone derivatives

#### 2.3.1. Single-crystal growth of paliperidone derivatives

The experimental details are similar to the procedures reported in the literature (Ou *et al.*, 2020 ▸; Su *et al.*, 2018 ▸; Chen *et al.*, 2023 ▸). In brief, a small quantity of the sample was melted on a round coverslip at a specified temperature until a small single crystal remained. This seed was allowed to grow to a specified size at a temperature near the *T*_m_ (0.97–0.98 *T*_m_) and was then submitted for SCXRD analysis.

#### 2.3.2. Growth kinetic measurement

The crystal growth rate of paliperidone derivatives in the melt was measured using a polarized light microscope (Olympus BX53) equipped with a THMS 600 hot stage (Linkam, UK). The sample (3–5 mg) was melted between two clean coverslips until no crystals remained. The resulting sandwiched sample was quenched at room temperature on an aluminium block to obtain a clear amorphous film. The seeds were placed in contact with the amorphous film to initiate crystal growth. The growth rate was measured by tracking the advance of the crystal front over time. The reported growth rate was obtained from at least three measurements for three independent samples.

#### 2.3.3. Viscosity measurement

The viscosities of amorphous paliperidone derivatives were measured at various temperatures using a HAAKE MARS 60 rheometer (Thermo Fisher Scientific, USA). Viscosity measurements were performed between two parallel plates 20 mm in diameter with a gap size of 0.3 mm. The crystalline powder of paliperidone derivatives was added and melted at 5°C above the *T*_m_ of the corresponding derivatives for 1 min to ensure no crystals remained. The system was then cooled to the desired temperature and isothermed for 2 min. Shear deformation was implemented at a rate of 1 s^−1^ with the temperature held constant, and the shear viscosity was recorded when the measurement reached a steady state.

### 2.4. Solution crystallization of paliperidone derivatives

#### 2.4.1. Induction time measurement

The solubility of paliperidone derivatives in iso­propanol at 20°C was determined by the gravimetric method prior to induction time measurement (experimental details and results are given in the supporting information). According to the solubility data, a saturated solution of paliperidone derivatives was prepared by heating the desired amount of solid phase and solvent. The supersaturation was calculated as a mole fraction ratio (*S* = *x*/*x*_1_). The experimental method for induction time measurement follows the work of Jiang & Horst (2011 ▸). The saturated solution of paliperidone derivatives was filtered through a 0.22 µm nylon membrane, aliquoted into 15 preheated 10 ml glass vials and sealed immediately with poly(tetra­fluoro­ethyl­ene) (PTFE)-coated caps to minimize evaporation. The glass vial was reheated and stirred again at 50°C to dissolve the solid that crystallized from the process of filtration and dispensing. Then, the saturated solution was held at the nucleation temperature (20°C) for induction time measurement with a stirring speed at 400 rpm. The experimental temperature was controlled by a thermostatic water bath with an accuracy of ±0.01°C. Nucleation was observed by visual methods, and the time required for the solution to change from clear to cloudy was recorded as the induction time *t*. After nucleation, the vials were removed and reheated at 50°C to dissolve the crystals before the next nucleation experiment. The number of parallels in each set of experiments was 80–100. No solid phase transformation occurred during the nucleation study, which was confirmed by PXRD analysis (Fig. S2).

#### 2.4.2. Crystal growth rate measurement

Single crystals of paliperidone derivatives were obtained in iso­propanol (IPA) by slow solvent evaporation. Single crystals with a well developed facet were selected for crystal growth experiments. The crystal growth rate measurements were carried out in IPA at 20°C (*S* = 3). The saturated solution was prepared and filtered following the procedures used in the nucleation induction time measurements. Then, the filtrate was transferred to a growth tank for 15 min at 20°C to generate supersaturation. A single crystal of a paliperidone derivative was placed in the growth tank for the growth rate measurements. The crystal growth rates of paliperidone derivatives were measured using an inverted fluorescence microscope (ECLIPSE Ts2R, Nikon, Japan) at regular time intervals. The reported growth rate was obtained from six parallel sets of experiments.

## 3. Results and discussion

### 3.1. Solid-state characterization

**Figure 2:**
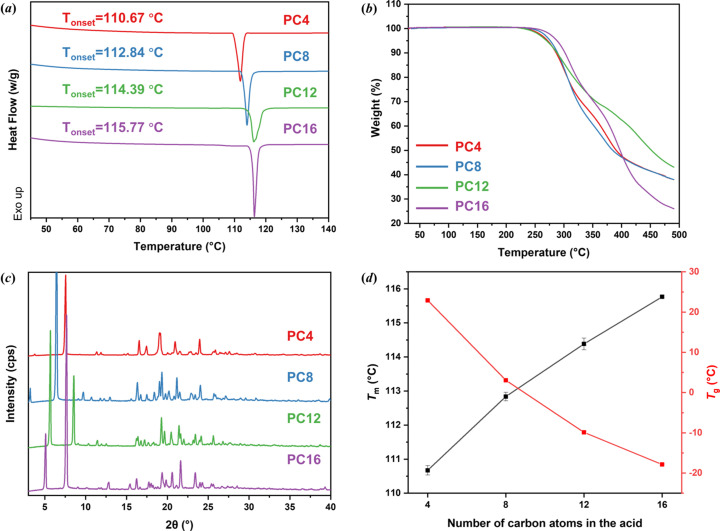
(*a*) DSC thermograms. (*b*) TGA thermograms. (*c*) PXRD diffraction patterns. (*d*) *T*_m_ and *T*_g_ of paliperidone derivatives.

The thermal properties of paliperidone derivatives were analyzed by DSC and TGA. A single distinct sharp endothermic peak appeared in the DSC trace of each paliperidone derivative [Fig. 2 ▸(*a*)], indicating high phase purity for all model compounds. The glass transition temperatures of the paliperidone derivatives are provided in Fig. S1. The melting point (*T*_m_) and glass transition temperature (*T*_g_) of paliperidone derivatives were plotted against the number of carbon atoms in their respective fatty acid fractions [Fig. 2 ▸(*d*)]. A paliperidone derivative with a longer alkyl chain length has a higher *T*_m_ and a lower *T*_g_. The TGA showed that all paliperidone derivatives have good thermal stability and that degradation occurs far above their *T*_m_ values [Fig. 2 ▸(*b*)]. The X-ray diffraction patterns of the paliperidone derivatives are similar [Fig. 2 ▸(*c*)] but there are distinct differences in the diffraction peaks present at low diffraction angles (<10°).

### 3.2. Crystal structure analysis

Thin plate-like single crystals of paliperidone derivatives (Fig. 5) were successfully obtained by the melt droplets method and studied by SCXRD. The crystallographic parameters of the paliperidone derivatives are shown in Table 1 ▸. The paliperidone derivatives crystallized isostructurally in the monoclinic system with the *P*2_1_/*c* space group regardless of the length of the alkyl chain, and each contains four molecules in the corresponding unit cell. With increasing length of alkyl chain, the unit-cell parameters *a* and *c* increase, whereas the *b* axis decreases.

**Figure 3:**
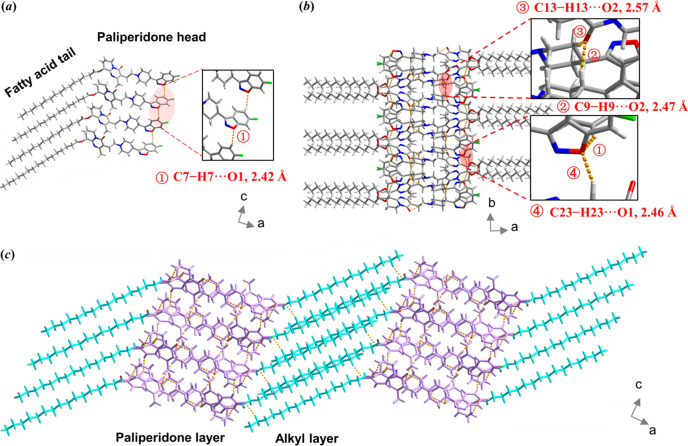
(*a*) 1D chain consisting of multiple PC16 molecules. (*b*) 2D packing of PC16. (*c*) Projection of the crystal structure of PC16.

Despite varied alkyl chain lengths, the paliperidone derivatives exhibit isostructural crystal packing, sharing the same conjugated backbones and crystal-packing motifs. The paliperidone derivative molecule consists of a paliperidone head and a fatty acid tail. In the case of PC16, as a representative example of the paliperidone derivatives, multiple PC16 molecules connect with each other by the hydrogen on the phenyl ring and the oxygen on isoxazole (C7—H7⋯O1: 2.42 Å) stacking along the *c* axis, forming a 1D chain [Fig. 3 ▸(*a*)]. These 1D chains are stacked along the *b* axis by C—H⋯O hydrogen bonding interactions (C9—H9⋯O2: 2.47 Å, C13—H13A⋯O2: 2.57 Å, C23—H23⋯O1: 2.46 Å), leaving the paliperidone heads aligned and the fatty acid tails opposite and parallel, forming the 2D layer of PC16 [Fig. 3 ▸(*b*)]. This 2D layer is further extended by C—H⋯F interactions (C39—H39A⋯F1: 2.51 Å) which consequently form a lamellar structure of two segregated layers: an alkyl layer and a paliperidone layer [Fig. 3 ▸(*c*)].

**Figure 4:**
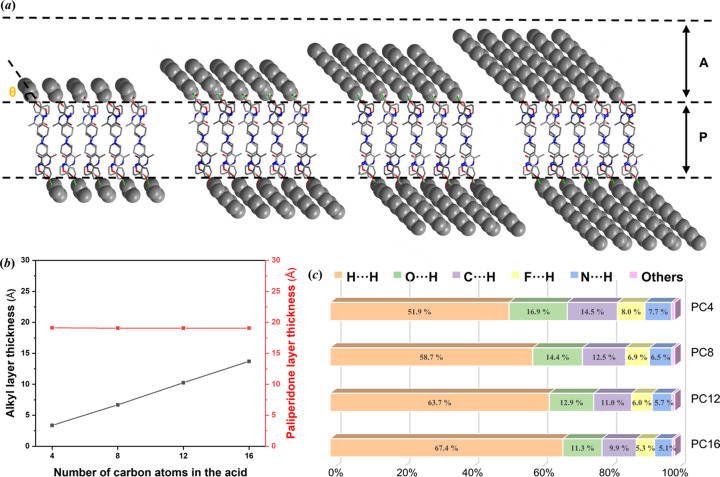
(*a*) Molecular packing structures of paliperidone derivatives. (*b*) Alkyl layer and paliperidone layer thicknesses of the paliperidone derivatives. (*c*) Summary of the various contact contributions in the paliperidone derivatives.

The thickness of the alkyl layer (represented by A in Fig. 4 ▸) significantly increases with increasing number of carbon atoms in the paliperidone derivatives, whereas the thickness of the paliperidone layer (represented by P in Fig. 4 ▸) remains constant [Fig. 4 ▸(*a*)]. The elongated straight alkyl chains of the molecules are almost parallel and tilted from the *b* *c* plane. The tilt angle decreases with increasing alkyl chain length in the derivatives, with the ranking θ_PC4_(62.52°) > θ_PC8_(49.39°) > θ_PC12_(47.68°) > θ_PC16_(47.37°).

Hirshfeld surfaces analysis, an effective tool to investigate and visualize the intermolecular interactions in the crystal, was performed on the paliperidone derivative crystals (Spackman & McKinnon, 2002 ▸; Spackman & Jayatilaka, 2009 ▸; He *et al.*, 2015 ▸). 2D fingerprint plots are provided in Fig. S3. A detailed analysis of the relative contributions of the intermolecular contacts is shown in Fig. 4 ▸(*c*). The H⋯H interaction made the largest contribution, suggesting that van der Waals interactions are the dominant interactions in paliperidone derivative crystals, and the relative contribution increases with increasing alkyl chain length due to the longer alkyl chains providing a higher proportion of hydrogen atoms in the structure (Martin *et al.*, 2015 ▸). In addition, the paliperidone derivatives also connect with each other by weak hydrogen bonds (Table S2). In contrast to van der Waals interactions, the relative contribution of hydrogen-bonding interactions decreases with increasing alkyl chain length.

### 3.3. Crystal morphology in melt and solution

**Figure 5:**
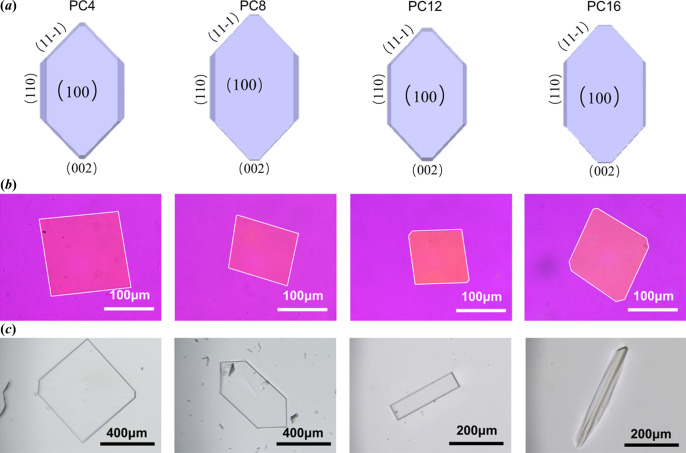
(*a*) Predicted crystal morphologies of paliperidone derivatives and the experimental morphologies grown from (*b*) the melt and (*c*) solution.

The Bravais–Friedel–Donnay–Harker (BFDH) model was applied for the crystal morphology prediction of the paliperidone derivatives. It revealed that the predicted crystal habits of the paliperidone derivatives are quite similar and the (100) faces are predominant in the crystals regardless of the length of the alkyl chain [Fig. 5 ▸(*a*)]. Figs. 5 ▸(*b*) and 5 ▸(*c*) show the crystals of paliperidone derivatives obtained from the melt and solution crystallization, respectively. The morphologies of crystal growth of paliperidone derivatives over time are provided in Fig. S4. It can be seen that the experimental morphology does not fit well with the predicted morphology. The difference can be rationalized because the BFDH model is mainly based on lattice parameters and symmetry; in fact, crystal morphology is controlled by both internal structure and crystallization conditions. In general, the crystals grown from the melts were very thin and plate-like with predominant (100) faces, and the morphology is almost the same independent of alkyl chain length. Conversely, the crystal morphology of paliperidone derivatives crystallized in IPA is quite different with respect to alkyl chain length. PC4 and PC8, the derivatives with shorter alkyl chains, appeared to grow as plate-shaped crystals with smaller aspect ratios. PC12 and PC16, derivatives with longer alkyl chains, tended to grow as rod-like crystals with larger aspect ratios. The distinct difference in crystal morphology of the paliperidone derivatives from solution crystallization may be caused by the solvent–solute interactions between the solvent and the crystal facets, which are absent in the melt (Ji *et al.*, 2022 ▸).

### 3.4. Crystallization kinetics of paliperidone derivatives in the melt

**Figure 6:**
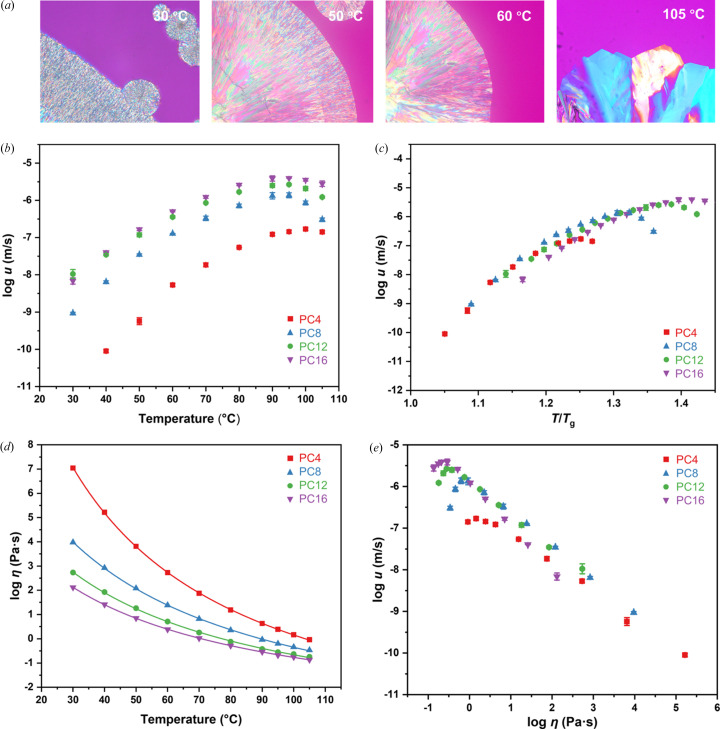
(*a*) Crystal growth morphologies of PC16 crystals in the melt at different temperatures. (*b*) Melt crystallization kinetics of paliperidone derivatives. (*c*) Crystal growth rates of paliperidone derivatives in the melt as a function of *T*/*T*_g_. (*d*) Viscosity of paliperidone derivatives (solid line represents VFT equation-fitting curves). (*e*) Crystal growth rate plotted against viscosity for paliperidone derivatives.

The paliperidone derivatives show distinctive crystallization behavior in the melt. The crystallization tendency increases with alkyl chain length. At room temperature, PC16 crystallized within one day, PC12 and PC8 crystallized after three days, and the crystallization was much more difficult for PC4 (Fig. S5). The crystal growth kinetics of paliperidone derivatives in the melt were determined by seeding crystallization (for details, see Methods[Sec sec2]) through tracking the advance of the crystal front between two coverslips at set time intervals. Fig. 6 ▸(*a*) shows the morphologies of PC16 crystals grown over a wide range of temperatures from 30 to 105°C. At the temperature approaching the melting point of PC16 crystals (*e.g.* 105°C), the crystals grew as single crystals. When the liquid was cooled to 60°C, the product was a fine-grained polycrystalline material. Compact spherulites were observed when grown at relatively low temperatures (*e.g.* 30°C).

The crystal growth rates of paliperidone derivatives as a function of temperature (from 30 to 105°C) are overlaid in Fig. 6 ▸(*b*). The growth kinetics of the paliperidone derivatives between 30 and 105°C exhibit characteristic bell-shaped curves, as a result of the competition between molecular mobility and the thermodynamic driving force for the crystallization (Zhang *et al.*, 2021 ▸; Shi & Cai, 2016 ▸). The order of growth rate of paliperidone derivatives is PC16 ≃ PC12 > PC8 ≫ PC4. However, the four paliperidone derivatives have similar crystal growth rates within an order of magnitude when compared at the same temperature relative to *T*_g_ [Fig. 6 ▸(*c*)]. Additionally, the viscosity of the supercooled liquid of paliperidone derivatives [Fig. 6 ▸(*d*)] decreases with increasing alkyl chain length at the same temperature. This order was consistent with the order of paliperidone derivative crystal growth rates. Fig. 6 ▸(*e*) shows the growth rates of paliperidone derivatives as a function of liquid viscosity in the form of a log–log format, where a linear correlation was present, suggesting liquid viscosity contributes to the differences in growth rates of the four paliperidone derivatives. The slight deviation at temperatures close to *T*_m_ can be attributed to the fact that crystal growth is mainly limited by the thermodynamic driving force in that temperature region. Generally, the kinetic contributions of crystal growth depend on the molecular diffusion. According to the Stokes–Einstein equation, the diffusion coefficient is inversely related to the viscosity η for weakly supercooled liquids, whereas decoupling of diffusion and viscosity occurs at temperatures close to *T*_g_ (at *T* < 1.3 *T*_g_) (Grzybowska *et al.*, 2016 ▸). Thus, the growth rate depends on the viscosity of the supercooled liquid at a temperature far above *T*_g_, wherein molecules diffuse faster with lower liquid viscosities, and thus kinetically favor crystal growth. It is reasonable that PC4, which has the shortest chain length, grows the slowest at the same temperature due to its higher viscosity. As the chain length increases, the viscosity of the paliperidone derivative liquid decreases, leading to increased molecular mobility, and thus the growth rate increases. This trend is evident from C4 to C8. However, the viscosity or mobility is less affected as the chain length increases further, and the difference in growth rate between PC12 and PC16 is relatively small.

### 3.5. Crystallization kinetics of paliperidone derivatives in solution

**Figure 7:**
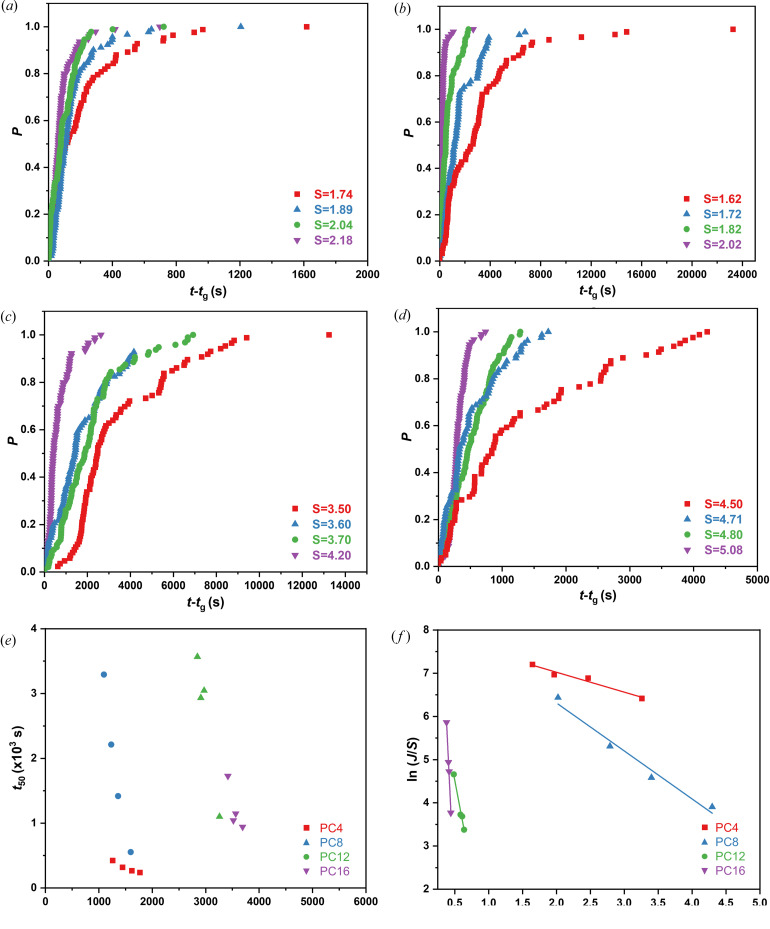
Induction time distributions of (*a*) PC4, (*b*) PC8, (*c*) PC12 and (*d*) PC16 at different supersaturations in IPA at 20°C. (*e*) Nucleation times versus the thermodynamic driving force in IPA at 20°C. (*f*) ln(*J*/*S*) versus 1/ln^2^ *S* for the induction time measurements enables the determination of the nucleation parameters *A* and *B* from equation (3).

Solution crystallization is widely used for separation and purification of pharmaceutical compounds. It is of interest to investigate the effect of alkyl chain length on the crystallization behavior of paliperidone derivatives in solution. The study of nucleation induction times was conducted as a function of supersaturation at 20°C in IPA, a commonly used solvent for the synthesis of paliperidone derivatives. Due to the stochastic nature of nucleation, over 1200 isolated experiments were carried out for all paliperidone derivatives, the measured induction times can be approximated to a cumulative probability distribution *P*(*t*):

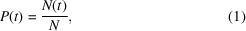

where *N* is the number of isolated experiments and *N*(*t*) is the number of experiments in which crystals are detected at time *t*. According to the Poisson distribution, the nucleation rate (*J*) is given by

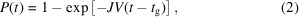

where *V* is the solution volume and *t*_g_ is the detection time delayed by crystal growth, which can be approximated as the minimum induction time observed under each supersaturation. Fitting equation (2) to the experimental probability distribution *P*(*t*) can lead to a value for the nucleation rate (*J*) and the growth time (*t*_g_) at a given supersaturation. The probability distributions *P*(*t*) of induction time of the paliperidone derivatives in IPA at different supersaturations are shown in Figs. 7 ▸(*a*)–7(*d*). As expected, the induction time *t* decreases with increasing degree of supersaturation *S*. To evaluate the difficulty of nucleation among the paliperidone derivatives, the median induction times (*t*_50_) were extracted from the experimental induction time distribution (McTague & Rasmuson, 2023 ▸). Fig. 7 ▸(*e*) illustrates the correlations of the median nucleation time of the paliperidone derivatives against the required driving force. The results show that the nucleation of PC16 is the most difficult in IPA, followed by PC12, PC8 and PC4. That is, the paliperidone derivatives nucleate with more difficultly with a longer chain length.

According to classical nucleation theory, the nucleation rate *J* can be described as

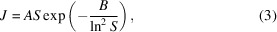

where *A* and *B* represent nucleation kinetic and thermodynamic parameters, respectively, and *S* is the supersaturation. The kinetic parameter *A* reflects the attachment rate of solute molecules to the nuclei. The thermodynamic parameter *B* is related to the energy barrier that needs to be overcome to form nuclei from solution. The thermodynamic parameter *B* can be defined as

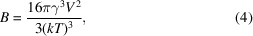

where γ is the solid–liquid interfacial energy per unit area, *V* is the molecular volume, *T* is the nucleation temperature and *k* is the Boltzmann constant. The parameters *A* and *B* could be determined using a plot of ln(*J*/*S*) against ln^−2^ *S* [Fig. 7 ▸(*f*)], and from the latter, the interfacial energy γ is determined. The *A* and γ parameters determined for the paliperidone derivatives in IPA are listed in Table 2 ▸. The value of the nucleation kinetic parameter *A* increases with increasing the alkyl chain length of paliperidone derivatives in IPA, and the value of the nucleation kinetic parameter *A* of PC16 is several orders of magnitude larger than that of PC4 in the same solution. Conversely, the solid–liquid interfacial energy γ of the paliperidone derivative crystals in IPA decreases with increasing alkyl chain length in the order PC16 > PC12 > PC8 > PC4. The solute–solvent interaction energy is reported to be proportional to γ, and stronger interactions enable higher energy barriers for nuclei formation from solution (Chai *et al.*, 2019 ▸). The overall solute–solvent interaction energies were calculated from molecular dynamics simulations, showing the following order (see Table 3 ▸ and the supporting information for details): PC4 < PC8 < PC12 < PC16, which is consistent with the order of magnitude of the solid–liquid interfacial energy γ derived from classical nucleation theory as expected. Given the relation between the induction time and alkyl chain length determined above, the nucleation behavior of paliperidone derivatives is dominated by thermodynamics within the supersaturation range investigated rather than kinetics, suggesting that overcoming the interfacial energy barrier is the rate-limiting process for nucleation of the paliperidone derivatives.

**Figure 8:**
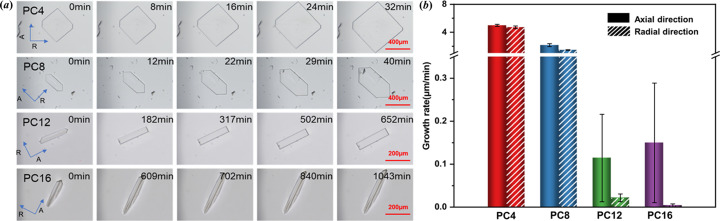
(*a*) Crystal growth morphology of paliperidone derivatives in IPA. (*b*) Growth rates of paliperidone derivatives in IPA at 20°C (histograms: growth rate in axial direction, striped histograms: growth rate in the radial direction).

Crystal growth experiments of the paliperidone derivatives in solution were conducted at high supersaturation (*S* = 3) because PC16 grew too slowly in IPA at low supersaturation. We determined the growth rate of the axial [represented by A in Fig. 8 ▸(*a*)] and radial [represented by R in Fig. 8 ▸(*b*)] directions of the crystals. The morphologies and growth rates are summarized in Figs. 8 ▸(*a*) and 8 ▸(*b*). The results reveal that all paliperidone derivatives crystals grow faster in the axial direction than in the radial direction and the order of the growth rate of paliperidone derivatives in IPA is PC4 > PC8 ≫ PC12 ≃ PC16 in both the axial and the radial directions. The order of growth rates of paliperidone derivatives in IPA is consistent with the order of the nucleation rates. According to classical nucleation theory, the clusters are assumed to have a similar structure and morphology to the mature crystal. The cluster growth rate will be consistent with or proportional to the crystal growth rate (Kashchiev, 2000 ▸). Thus, it can be rationalized that the fastest growth rate of the PC4 molecules contributes to the fastest nucleation rate among paliperidone derivatives (Cruz-Cabeza *et al.*, 2017 ▸; Liu *et al.*, 2019 ▸).

## 4. Conclusions

In summary, the role of alkyl chain length for a series of fatty acid-derivative paliperidone prodrugs in the crystallization behavior in both melt and solution was investigated. The paliperidone aliphatic derivatives showed isostructural crystal packing, a characteristic lamellar structure, and the crystals exhibited the dominant (100) face in both melt and solution crystallization, which are independent of the alkyl chain length. It revealed that paliperidone derivatives are more difficult to crystallize with a shorter chain length in the melt, but the trend in solution is the opposite. The relation between the alkyl chain length and the crystallization tendency of the paliperidone derivatives in melts is mainly associated with molecular mobility. In contrast, the interfacial energy dominates the crystallization of paliperidone derivatives in solution. This work provides insights into the relation between alkyl chain length in fatty acid-based prodrugs and crystallization behavior, deepening our understanding of the correlation between molecular structure and crystallization. It is also relevant to predict the crystallization behavior of derivatives with varied alkyl chain lengths, which in turn guides the prodrug design and the development of prodrug-based extended-release formulations.

## Supplementary Material

Crystal structure: contains datablock(s) PC4, PC8, PC12, PC16. DOI: 10.1107/S2052252523009582/lq5053sup1.cif

Structure factors: contains datablock(s) PC4. DOI: 10.1107/S2052252523009582/lq5053PC4sup2.hkl

Structure factors: contains datablock(s) PC8. DOI: 10.1107/S2052252523009582/lq5053PC8sup3.hkl

Structure factors: contains datablock(s) PC12. DOI: 10.1107/S2052252523009582/lq5053PC12sup4.hkl

Structure factors: contains datablock(s) PC16. DOI: 10.1107/S2052252523009582/lq5053PC16sup5.hkl

Supporting information, tables and figures. DOI: 10.1107/S2052252523009582/lq5053sup6.pdf

CCDC references: 2271248, 2271249, 2271250, 2271251

## Figures and Tables

**Table 1:** Crystallographic parameters of paliperidone derivatives

	PC4	PC8	PC12	PC16
Empirical formula	C_27_H_33_FN_4_O_4_	C_31_H_41_FN_4_O_4_	C_35_H_49_FN_4_O_4_	C_39_H_57_FN_4_O_4_
Formula weight	496.57	552.68	608.78	664.88
Crystal system	Monoclinic	Monoclinic	Monoclinic	Monoclinic
Space group	*P*2_1_/*c*	*P*2_1_/*c*	*P*2_1_/*c*	*P*2_1_/*c*
Temperature (K)	170	170	170	100
*a* (Å)	23.295 (4)	26.953 (11)	31.029 (3)	34.188 (3)
*b* (Å)	10.179 (2)	10.080 (4)	9.9381 (10)	9.8280 (9)
*c* (Å)	10.6479 (19)	10.753 (4)	10.8384 (11)	10.8068 (9)
α (°)	90	90	90	90
β (°)	97.284 (6)	92.658 (12)	99.374 (3)	92.992 (3)
γ (°)	90	90	90	90
*V* (Å^3^)	2504.4 (8)	2918 (2)	3297.6 (6)	3626.1 (5)
*Z*	4	4	4	4
ρ_calc_ (g cm^−3^)	1.317	1.258	1.228	1.218
*R*_1_, w*R*_2_ [*I* > 2σ(*I*)]	0.0974, 0.2334	0.0827, 0.1807	0.059, 0.1112	0.0556, 0.1165
*R*_1_, w*R*_2_ (all data)	0.1746, 0.2707	0.1921, 0.2323	0.1392, 0.1483	0.1155, 0.1462
CCDC No.	2271248	2271249	2271250	2271251

**Table 2:** Nucleation parameters derived from classical nucleation theory

	PC4	PC8	PC12	PC16
*A* (m^−3^ s^−1^)	2.8 × 10^3^	5.1 × 10^3^	7.6 × 10^3^	8.3 × 10^7^
γ (mJ m^−2^)	1.67	2.02	3.64	5.40

**Table 3:** Solvent–solute interaction energies calculated from molecular dynamics simulations at 20°C Data are presented as means ± the standard deviation (*n* = 10).

Solute + solvent	PC4 + IPA	PC8 + IPA	PC12 + IPA	PC16 + IPA
Δ*E*_int_ (kJ mol^−1^)	−267.96 ± 13.71	−295.89 ± 5.86	−439.54 ± 6.78	−486.73 ± 5.92
