# Supplementary material for: The role of alkyl chain length in the melt and solution crystallization of paliperidone aliphatic prodrugs
Source: IUCrJ. 2024 Jan 1;11(Pt 1):23–33. doi: 10.1107/S2052252523009582 (PMC10833388; doi:10.1107/S2052252523009582)
Supplement: Supplementary file 6 [file m-11-00023-sup6.pdf]

IUCrJ

Volume 11 (2024)

Supporting information for article:

The role of alkyl chain length in the melt and solution crystallization of paliperidone aliphatic prodrugs

An Chen, Peishan Cai, Yayun Peng, Minshan Guo, Yuan Su and Ting Cai

S1. Experiments

S1.1. Solubility measurement

The solubility of paliperidone derivatives in IPA at 20 °C was measured by gravimetric method. The excess amount of paliperidone derivatives was added to a 20 mL round-bottomed flask containing 10mL, which was stirred in a water bath at 20 °C. After 24 h, the stirring was stopped, and the system was allowed to stand for 2 h. The supernatant was filtered through a 0.22 μm filter into a pre-weighed 20 mL glass vial, and the total weight was recorded. Then the glass vial was transferred into fume hood to evaporate the solvent. Each experiment was repeated three times.

$$x_1 = \frac{m_1/M_1}{m_1/M_1 + m_2/M_2} \quad \text{eq (1)}$$

S1.2. Molecular dynamic simulation

All the calculations were performed in the Forcite module of *Material Studio* software. In short, the simulation can be divided into three steps. First, geometry optimization was performed on paliperidone derivatives and solvent molecules to minimize energy. Second, an amorphous cell was constructed and optimized. Third, the optimized amorphous cell was equilibrated by performing an NVT MD simulation at 293.15 K and 1 atm. The total simulation time was set as 500 ps with a step of 1 fs. RDF analysis was carried out based on the final crystal structure after the molecular dynamics trajectory. All simulations were implemented in the COMPASS II force field along with the force field assigned charges. The interaction energy between solute and solvent was calculated according to

$$\Delta E_{\text{interaction}} = E_{\text{total}} - (E_{\text{solute-solute}} + E_{\text{solvent-solvent}}) \quad \text{eq (2)}$$

Table S1

Solubility of paliperidone derivatives in IPA at 20 °C

Paliperidone derivatives	Solubility in mole fraction (x_1)
PC4	1.92*10^{-3}
PC8	4.03*10^{-4}
PC12	9.83*10^{-5}
PC16	3.42*10^{-5}

Table S2

Hydrogen bond informations for paliperidone derivatives

	No.	D−H···A	D−H	H···A	D···A	D−H···A	Symmetry code
PC4	1	C(3)-H(3B)···F(1)	0.99	2.54	3.390(8)	143	1-x,1/2+y,3/2-z
	2	C(5)-H(5)···O(1)	1.00	2.52	3.300(8)	134	1-x,-1/2+y,3/2-z
	3	C(17)-H(17A)···O(2)	0.99	2.53	3.466(8)	158	1-x,1-y,2-z
	4	C(18)-H(18)···O(2)	1.00	2.53	3.230(7)	127	1-x,-1/2+y,3/2-z
	5	C(24)-H(24)···O(1)	0.95	2.48	3.322(8)	148	x,1/2-y,1/2+z
	6*	C(7)-H(7A)···O(3)	0.99	2.57	2.933(9)	101	
	7*	C(15)-H(15A)···O(2)	0.99	2.58	3.147(8)	117	

	No.	D−H···A	D−H	H···A	D···A	D−H···A	Symmetry code
PC8	1	C(6)-H(6)…O(1)	0.95	2.48	3.313(6)	147	
	2	C(8)-H(8)···O(2)	1.00	2.53	3.215(6)	126	x,1/2-y,-1/2+z
	3	C(12)-H(12B)···O(2)	0.99	2.57	3.517(6)	160	1-x,-1/2+y,1/2-z
	4	C(20)-H(20)···O(1)	1.00	2.52	3.306(6)	135	1-x,1-y,-z
	5*	C(13)-H(13B)···O(2)	0.99	2.57	3.147(6)	117	x,1/2-y,-1/2+z
	6*	C(22)-H(22B)···O(3)	0.99	2.57	2.936(6)	102	

	No.	D−H···A	D−H	H···A	D···A	D−H···A	Symmetry code
PC12	1	C(4)-H(4)···O(1)	0.95	2.46	3.314(4)	148	x,3/2-y,-1/2+z
	2	C(8)-H(8)···O(2)	1	2.49	3.174(3)	126	1-x,1/2+y,1/2-z
	3	C(12)-H(12A)···O(2)	0.99	2.59	3.541(4)	161	1-x,1-y,-z
	4	C(20)-H(20)···O(1)	1	2.5	3.271(4)	133	1-x,1/2+y,1/2-z
	5*	C(13)-H(13A)···O(2)	0.99	2.57	3.150(4)	117	
	6*	C(22)-H(22A)···O(3)	0.99	2.58	2.941(4)	102	

	No.	D−H···A	D−H	H···A	D···A	D−H···A	Symmetry code
PC16	1	C(7)-H(7)…O(1)	0.95	2.42	3.390(8)	149	x,1/2-y,-1/2+z

2	C(9)-H(9)···O(2)	1	2.47	3.300(8)	125	1-x,-1/2+y,-1/2-z
3	C(13)-H(13A)···O(2)	0.99	2.57	3.466(8)	163	1-x,1-y,-1-z
4	C(23)-H(23)···O(1)	1	2.46	3.230(7)	132	1-x,-1/2+y,-1/2-z
5	C(39)-H(39A)···F(1)	0.99	2.51	3.322(8)	158	1+x,1/2-y,3/2+z
6 *	C(15)-H(15A)···O(2)	0.99	2.55	2.933(9)	117	
7 *	C(21)-H(21[a])···O(3)	0.99	2.58	3.147(8)	101	

*: Intramolecular hydrogen bond

Table S3

Nucleation rate of paliperidone derivatives in isopropanol at different supersaturation ratio

	S	J (m^{-3} s^{-1})	
PC4	1.74	1062	
	1.89	1850	$\ln (J/S) = -0.4661 / \ln^2 S + 7.9599$
	2.03	2163	$r^2=0.969$
	2.18	2924	
PC8	1.62	80	
	1.72	168	$\ln (J/S) = -1.1105 / \ln^2 S + 8.5311$
	1.82	368	$r^2=0.975$
	2.02	1259	
PC12	3.50	102	
	3.60	143	$\ln (J/S) = -8.3133 / \ln^2 S + 8.6752$
	3.70	153	$r^2=0.984$
	4.20	442	
PC16	4.50	195	
	4.71	530	$\ln (J/S) = -32.6475 / \ln^2 S + 18.2358$
	4.80	670	$r^2=0.996$
	5.08	1782	

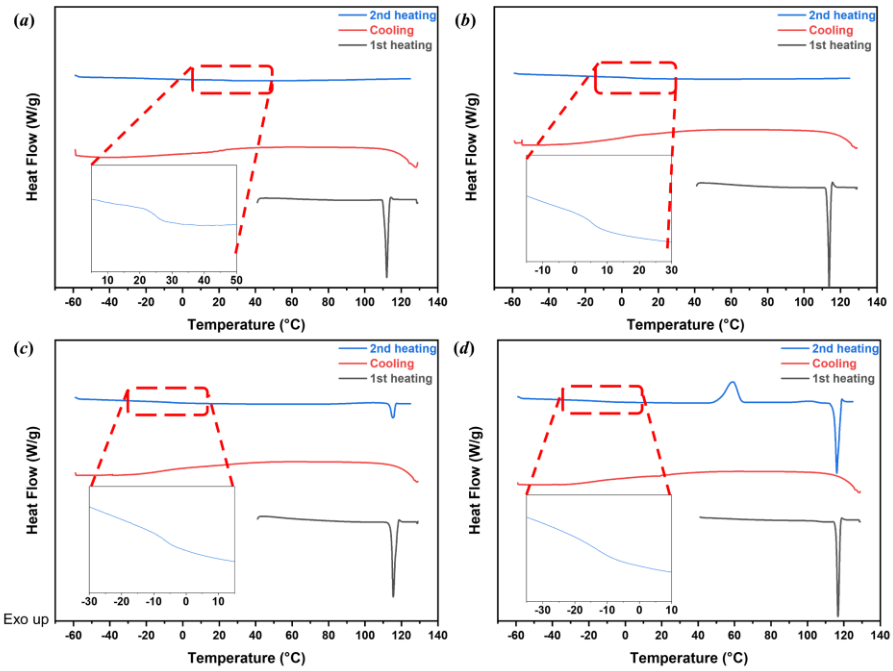

Figure S1

Determination of the Glass transition temperature (T_g) of (a) PC4, (b) PC8, (c) PC12, and (d) PC16 by DSC analysis.

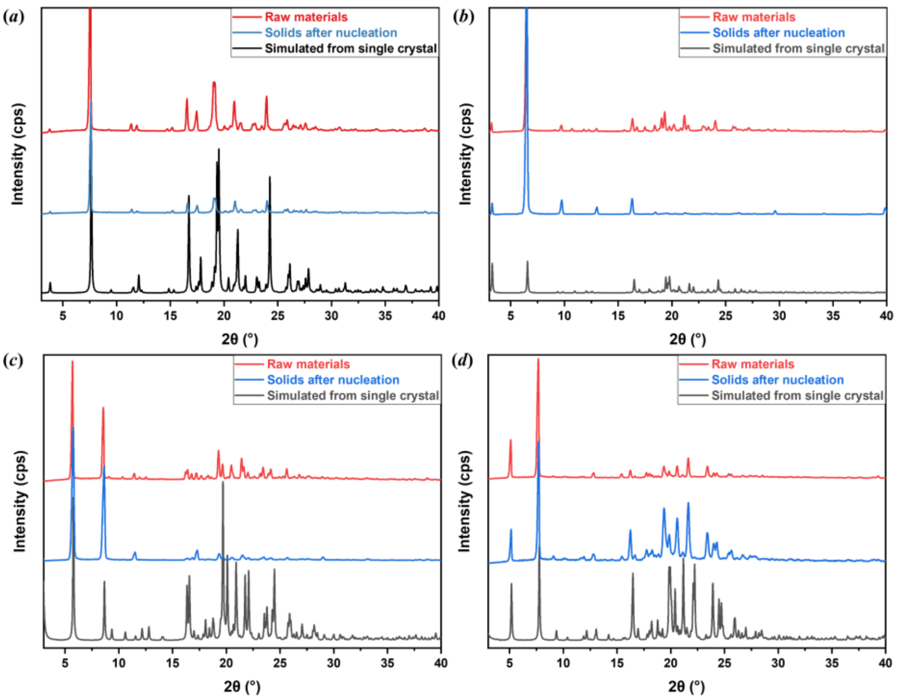

Figure S2

PXRD patterns of (*a*) PC4, (*b*) PC8, (*c*) PC12, and (*d*) PC16 after induction time measurement.

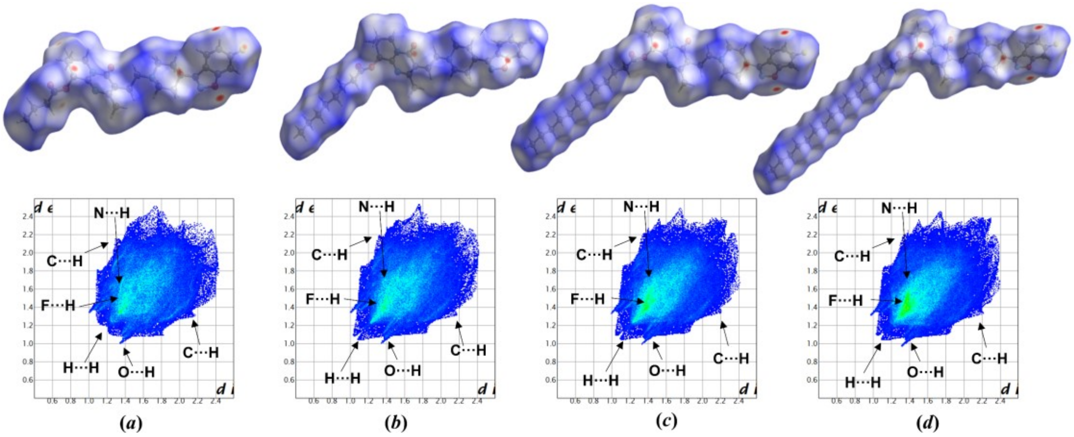

Figure S3

Hirshfeld surfaces (top) and 2D fingerprint plots (down) of (*a*) PC4, (*b*) PC8, (*c*) PC12, and (*d*) PC16.

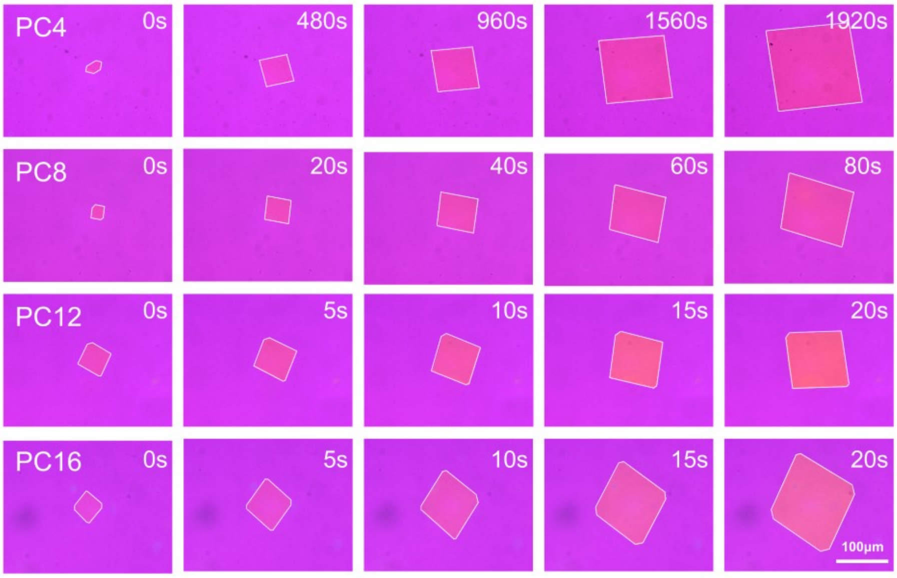

Figure S4

The morphologies of crystal growth of paliperidone derivatives over time.

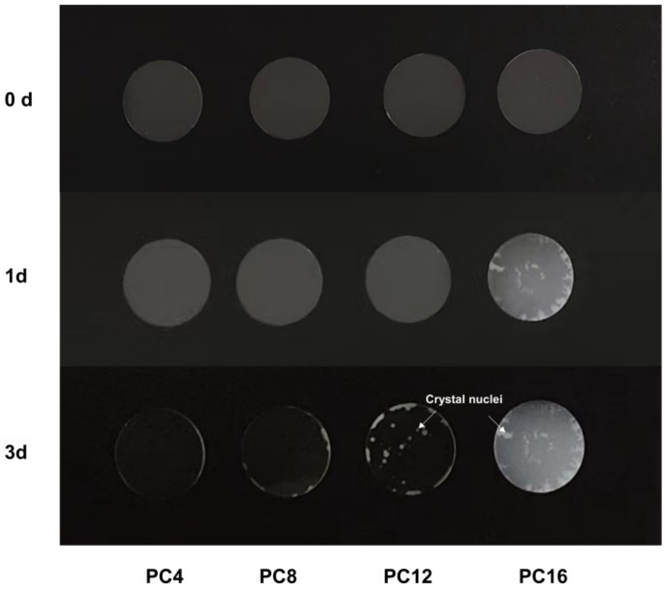

Figure S5

Crystallization behavior of paliperidone derivatives in melt at room temperature.

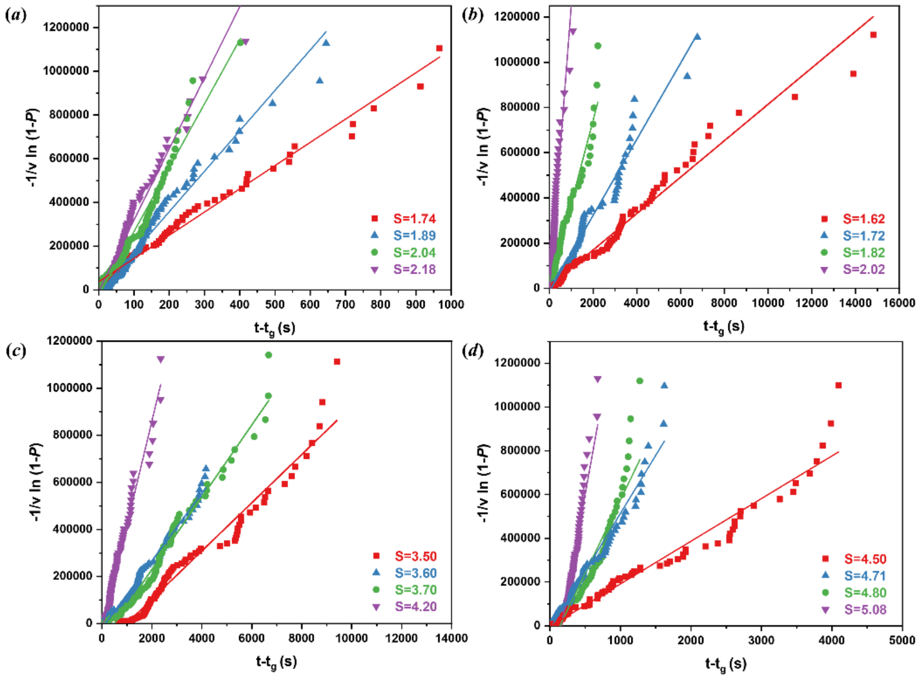

Figure S6

Induction time data plotted as −1/V ln(1 − P(t)) for paliperidone derivatives in isopropanol (a) PC4, (b) PC8, (c) PC12 and (d) PC16.
